# Knowledge and Practices on Antibiotic Use and Antibiotic Resistance Among Smallholder Pig Farmers in Timor-Leste

**DOI:** 10.3389/fvets.2021.819643

**Published:** 2022-01-06

**Authors:** Shawn Ting, Abrao Pereira, Steven Davis, Paulo Gabriel Vong da Silva, Amalia Alves, Cristibela Dos Santos, Jenny-Ann L. M. L. Toribio, Olavio Morais, Joanita Bendita da Costa Jong, Tamsin S. Barnes

**Affiliations:** ^1^Global and Tropical Health Division, Menzies School of Health Research, Charles Darwin University, Darwin, NT, Australia; ^2^Sydney School of Veterinary Science, Faculty of Science, University of Sydney, Camden, NSW, Australia; ^3^Ministry of Agriculture and Fisheries, Government of Timor-Leste, Dili, Timor-Leste; ^4^Epivet Pty. Ltd., Withcott, QLD, Australia; ^5^The University of Queensland, School of Veterinary Science, Gatton, QLD, Australia

**Keywords:** antibiotic use, knowledge, practices, Antimicrobial Resistance (AMR), smallholder, pig, Timor-Leste (East Timor), antimicrobials

## Abstract

Antibiotic resistance is an emerging global health threat which is linked to the overuse and misuse of antibiotics. This study was conducted to understand the knowledge and practices of smallholder pig farmers on antibiotic use and resistance in Timor-Leste. A cross-sectional study using a structured face-to-face interview was conducted in three municipalities. The interview was piloted and implemented in the local Tetun language. This study found that knowledge of antibiotics was very poor as only 12.7% (95% CI: 6.3–23.9) of farmers reported knowing what antibiotics were, and of these only one was able to correctly explain how an antibiotic worked. None of the farmers knew about antibiotic resistance and were able to explain the concept correctly. After the definition of antibiotic was explained to the farmer, only 3.6% (95% CI: 0.8–14.9) reported that their pigs had ever received antibiotics, and the majority of farmers whose pigs had not received antibiotics reported the lack of access to veterinary services. When used, antibiotics were only used for treatment with no reported use for disease prevention or growth promotion. None of the commonly used antibiotics were critically important antimicrobials. Compliance with withdrawal periods was not routinely followed. There is a need to improve access to government veterinary services for farmers in Timor-Leste, while addressing identified knowledge gaps on antibiotics and promoting prudent use practices. The findings from this study serve as baseline information to inform future interventions.

## Introduction

The rapid emergence of antibiotic resistance is a growing global threat that limits the effectiveness of antibiotic treatment which has been linked to the overuse and misuse of antibiotics (1, 2). In particular, several low and middle-income countries (LMICs) are reported to have high and inappropriate usage of antibiotics in the livestock sector driven by demand for animal protein and weak regulations (3–7). Unfortunately, LMICs are also more susceptible to the negative consequences of antibiotic resistance for human health due to a weaker health system and lack of alternative treatment strategies (8, 9).

Timor-Leste is a lower-middle income country (10) in Southeast Asia that shares a land border with Indonesia. At the time of the 2019 Agriculture Census, more agricultural households raised pigs than any other livestock species (81%) and the pig population was estimated at 4,53,444, second only to chickens (11). Pigs are also considered valuable animals with high cultural significance (12–14). Subsistence farming is the main livelihood for many rural households (15, 16) and most pigs are kept by smallholders (17), which is similar to many parts of Southeast Asia (18). Smallholder pig farms in Timor-Leste are low input and output systems (13, 19), although there are ongoing efforts to improve production (20, 21). Pigs are kept in permanent penning and/or tethering, semi-confined and free-roaming systems (19). Disease is the most commonly reported reason for pig mortality (19), and Timor-Leste experienced an African Swine Fever (ASF) outbreak in 2019 which caused high mortality in the pig population (22, 23). Other pig diseases such as Classical Swine Fever are endemic in Timor-Leste (24). The majority of veterinary services to agricultural households are provided by government-employed veterinary technicians and are free-of-charge (11, 25).

Studies investigating the knowledge and practices of antibiotic use and resistance among farmers have been conducted in some LMICs (7, 26). These studies mostly showed that antibiotic use by farmers was high despite a poor level of knowledge (27–30), and have been important for guiding interventions to promote prudent use of antibiotics to slow the emergence of resistance (31, 32). Such studies have never been conducted in Timor-Leste and would help characterise antibiotic knowledge and use at the farm level so appropriate strategies can be developed to avoid overuse and misuse, and facilitate implementation of the National Action Plan for Antimicrobial Resistance. Therefore, this study aims to describe the knowledge of smallholder pig farmers in Timor-Leste on antibiotics and antibiotic resistance and the antibiotic use practices in their pigs.

## Materials and Methods

### Study Period and Area

The study was conducted during August and September 2020 in three of the 13 municipalities in Timor-Leste: Liquica, Aileu, and Bobonaro. These three municipalities were selected based on proximity to the capital Dili and to the Indonesian border from where almost all veterinary antibiotics in the country are imported (25). According to the 2019 agriculture census, the number of pigs in Aileu, Bobonaro and Liquica were 14,896, 46,862 and 27,122 respectively (11). However, pig numbers may have reduced since the census due to mortality from ASF (22).

**Figure 1:**
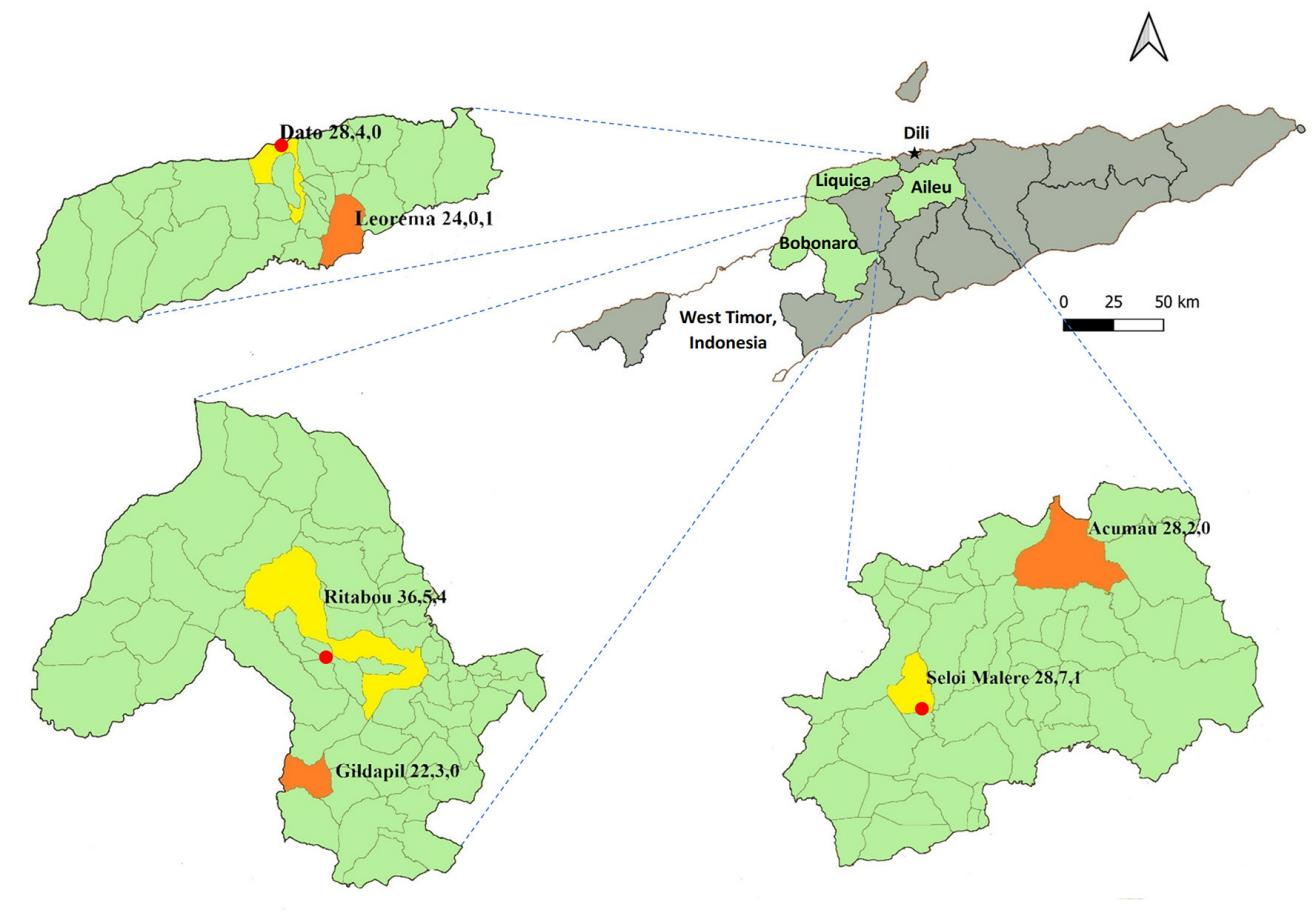
Map of Timor-Leste showing the three target municipalities (Liquica, Bobonaro, Aileu) and selected urban (yellow) and rural (orange) sucos. The red circle represents the capital of each municipality. Numbers shown for each suco refer to the number of farmers interviewed, the number of farmers who reported knowing what antibiotics are, and the number of farmers who reported that antibiotics had been used in their pigs, respectively.

Municipalities in Timor-Leste are sub-divided in sucos (villages). Due to resource and time constraints, one urban suco (near the municipal capital town) and one rural suco (far from the municipal capital town) were selected within each municipality based on road accessibility, community cooperation and a larger pig population. Figure 1 shows the sucos that were selected within each of the three municipalities.

### Study Design and Sampling

This study focused on pig farmers because pigs were commonly owned by households and have high socio-cultural and economic significance (13, 14). The target population was limited to smallholder pig farmers since most pigs are kept by smallholders (17). The eligibility criteria for farmer inclusion in the study were (1) keeping no more than 10 sows, (2) having kept pigs for at least one year in the last three years and (3) being involved in pig management decisions.

The required sample size was estimated using the online Epitools sample size calculator for estimating a proportion (33). The following assumptions were used: estimated prevalence of 50%, 8% desired precision and 95% confidence level. An estimated prevalence of 50% was used as there was no available estimate on the prevalence of antibiotic knowledge among farmers and use in pig herds. Assuming no clustering by suco, the minimum sample size required was 151, and this was divided equally between the six sucos. As there was no sampling frame available, participants in each suco were selected with the help of a local guide who was familiar with the agricultural activities in each household. Participants were selected from the area closest to the centre of the suco until the required sample size was achieved.

### Data Collection

The questionnaire was developed in English followed by translation into Tetun by one of the authors who is bilingual in English and Tetun. The questionnaire was piloted with 10 pig farmers and amended for clarity before implementation. The final version had three sections and contained mostly closed-ended questions with some open-ended questions. The responses to most open-ended questions were categorised retrospectively. The first section collected demographic information such farmer age, gender, education level, number of pigs owned, type of pig keeping methods, and their main income source. The second section assessed farmer knowledge on antibiotics and antibiotic resistance. Two questions in this section were asked as open-ended questions but immediately categorised by the interviewer (How do you think an antibiotic works and what is the impact of antibiotic resistance?). Following these questions that assessed if farmers had an accurate understanding of antibiotics and antibiotic resistance, the definition of antibiotics was clearly explained before proceeding to the third section of the questionnaire which assessed antibiotic use in pigs. Any responses offered by a participant that was in addition to the question asked was captured in free-text format. The English version of the questionnaire is available in the Supplementary Material.

The questionnaire was conducted face-to-face in Tetun by two trained Timorese veterinarians, and took an average of 15 minutes to complete. All responses were recorded on paper and subsequently entered and managed using REDCap electronic data capture tools hosted at Menzies School of Health Research (34, 35). All entries were checked for transcription errors by at least two members of the research team.

### Data Management and Analysis

Free text responses to two open-ended questions (main source of income, reasons for not using antibiotics) were categorised based on sets of categories developed retrospectively by three members of the research team. Descriptive analyses were conducted using Stata 17.0 (36). Categorical variables were described using absolute and relative frequency with 95% confidence intervals (95% CI) estimated for key parameters adjusted for clustering by suco. Continuous variables were summarised using mean and standard deviation and/or median and interquartile range (IQR).

## Results

### Farmer Demographics

A total of 165 farmers were interviewed, with 55 from Aileu, 58 from Bobonaro and 52 from Liquica municipality. Less than 10 farmers refused to participate in the interviews, although a precise number was not recorded. The reasons given for refusal were lack of time or interest. Slightly more than half of the participants lived in an urban suco (55.2%), and there were more female participants (56.4%). Mean age was 43.1 years (s.d. 15.3). Crop production was the most commonly reported main income source (37.0%) and few reported pig production as their main income source (8.5%). Farmer demographics are shown in Table 1.

**Table 1:** Demographic and pig raising information of 165 pig farmers (overall and by municipality) surveyed in Timor-Leste in 2020.

**Attribute**	**No. (%)**			
	**Aileu**	**Bobonaro**	**Liquica**	**Total**
	**(*n* = 55)**	**(*n* = 58)**	**(*n* = 52)**	**(*n* = 165)**
Location				
Urban	27 (49.1)	36 (62.1)	28 (53.8)	91 (55.2)
Rural	28 (50.9)	22 (37.9)	24 (46.2)	74 (44.8)
Gender				
Male	29 (52.7)	19 (32.8)	24 (46.2)	72 (43.6)
Female	26 (47.3)	39 (67.2)	28 (53.8)	93 (56.4)
Age (years)				
<30	6 (10.9)	11 (19.0)	22 (42.3)	39 (23.6)
34–44	18 (32.7)	17 (29.3)	12 (23.1)	47 (28.5)
45–60	20 (36.4)	24 (41.4)	13 (25.0)	57 (34.5)
> 60	11 (20.0)	6 (10.3)	5 (9.6)	22 (13.3)
Education				
None	15 (27.3)	15 (25.9)	8 (15.4)	38 (23.0)
Primary	14 (25.5)	9 (15.5)	10 (19.2)	33 (20.0)
Secondary	21 (38.2)	24 (41.4)	26 (50.0)	71 (43.0)
Post-secondary	5 (9.1)	10 (17.2)	8 (15.4)	23 (13.9)
Main incomes^a^				
Coffee production	4 (7.3)	5 (8.6)	22 (42.3)	31 (18.8)
Crop production except coffee	23 (41.8)	30 (51.7)	8 (15.4)	61 (37.0)
Livestock production (except pig) or fishing	4 (7.3)	1 (1.7)	9 (17.3)	14 (8.5)
Pig production	3 (5.5)	0 (0.0)	11 (21.2)	14 (8.5)
Public servant/suco administration	10 (18.2)	14 (24.1)	7 (13.5)	31 (18.8)
Pension	6 (10.9)	2 (3.4)	3 (5.8)	11 (6.7)
Small business/private sector	11 (20.0)	15 (25.9)	13 (25.0)	39 (23.6)
Casual labour	2 (3.6)	1 (1.7)	3 (5.8)	6 (3.6)
No information provided	1 (1.8)	0 (0.0)	0 (0.0)	1 (0.6)
Raising pigs at time of survey				
Yes	44 (80.0)	46 (79.3)	47 (90.4)	137 (83.0)
No	11 (20.0)	12 (20.7)	5 (9.6)	28 (17.0)
Pig management^a^				
Free-roaming	2 (3.6)	2 (3.4)	6 (11.5)	10 (6.1)
Tethered	33 (60.0)	7 (12.1)	27 (51.9)	67 (40.6)
Housed	27 (49.1)	50 (86.2)	31 (59.6)	108 (65.5)

a *Multiple responses allowed*.

### Farm Characteristics

At the time of the survey, 137 farmers (83.0%) were raising pigs. Of these, the median number of mature pigs (sows, boars and castrated males) kept was 1 (IQR: 1–2, range 0–10) and mean 1.5 (s.d. 1.3). The median number of young pigs (≤ 12 months) was 0 (IQR: 0–1, range 0–15) and mean 1.0 (s.d. 1.9). All but one of the 28 farmers who did not have pigs at that time reported that all their pigs had died in 2019 or 2020, with some specifically mentioning a disease outbreak in their neighbourhood. Two other farmers also reported recent deaths among their pigs with one farmer in Bobonaro reporting 20 deaths and one in Liquica reporting 19 deaths. Most farmers, particularly those in Bobonaro, kept at least some of their pigs housed. Only 10 farmers allowed their pigs to free-roam, of these four were from urban sucos and six from rural sucos. Of these, four farmers had exclusively free-roaming pigs.

### Knowledge of Antibiotics and Antibiotic Resistance

Few farmers reported knowing what antibiotics are (*n* = 21, 12.7% [95% CI: 6.3–23.9]). Of these, the most commonly mentioned sources of knowledge were friends and veterinary technicians. When specifically asked about how an antibiotic worked, only one (4.7%) of the 21 farmers correctly explained that antibiotics “kill or inhibit bacteria.” Amongst the same 21 farmers, only one (4.7%) reported hearing about antibiotic resistance but explained incorrectly that antibiotic resistance made antibiotics more effective, rather than less effective.

### Antibiotic Use Practices

Antibiotic use practices among smallholder pig farmers are detailed in Table 2. Few farmers (*n* = 6, 3.6% [95% CI: 0.8–14.9]) were confident that their pigs had been given antibiotics, of these four were from Ritabou, the urban suco in Bobonaro which is close to Indonesia. Most farmers responded that neither they nor anyone else had given their pigs antibiotics (*n* = 121, 73.3% [95% CI: 54.9–86.1]). The most common reason provided was no access to veterinary services. The remaining farmers (*n* = 38, 23.0% [95% CI: 11.9–38.9]) reported that they did not know whether their pigs received antibiotics, and the most common reported reason was that the animal received an injection but the farmer was unsure of the injected content. However, four of these 38 farmers indicated that their pigs had received treatment from technicians when sick so it was considered likely that they had been given antibiotics.

**Table 2:** Practices on antibiotic use on smallholder pig farms in Timor-Leste in 2020.

**Question**	**No. (%)**
Do you or anybody else give your pigs antibiotics? (*n* = 165)	
Yes	6 (3.6)
No	121 (73.3)
Don't know	38 (23.0)
If no, why not?^a^ (*n* = 121)	
Perceive antibiotic treatment is not effective	1 (0.8)
Not aware of veterinary services or antibiotics provided by government	69 (57.0)
No access to veterinary services	77 (63.6)
No access to antibiotics	7 (5.8)
Prefer other treatment options	4 (3.3)
Pigs are healthy/just started raising pigs	8 (6.6)
Pigs died too suddenly and unable to inform veterinary services	2 (1.7)
Not a meaningful response	4 (3.3)
No response	3 (2.5)
If yes, why do you use antibiotics? (*n* = 6)	
Treat disease	5 (83.3)
Prevention of disease	0 (0.0)
Promote growth	0 (0.0)
No response	1 (16.7)
If yes, what signs in animals will prompt you to use antibiotics?^a^ (*n* = 6)	
Diarrhoea	3 (50.0)
Fever	1 (16.7)
Respiratory signs	3 (50.0)
Skin infection	1 (16.7)
Other (erect hair coat, salivation, shivering)	1 (16.7)
If yes, does your pig feed contain antibiotics? (*n* = 6)	
Yes	0 (0.0)
No	4 (66.7)
No response	2 (33.3)
If yes, do you add antibiotics to water? (*n* = 6)	
Yes	0 (0.0)
No	4 (66.7)
No response	2 (33.3)
If yes, have your pigs ever been injected with antibiotics? (*n* = 6)	
Yes	5 (83.3)
No	0 (0.0)
No response	1 (16.7)
If injected, who injects the pigs? (*n* = 5)	
Yourself	2 (40.0)
Veterinary technician	2 (40.0)
Extension worker	1 (20.0)
If you inject, are the label instructions followed when using antibiotics? (*n* = 2)	
Yes	1 (50.0)
No	0 (0.0)
No response	1 (50.0)
Which antibiotics do you commonly use?^a^ (*n* = 6)	
Penstrep^b^	0 (0.0)
Medoxy^c^	4 (66.7)
Sulfastrong and/or Sulfabac^d^	1 (16.7)
Other	0 (0.0)
No information	2 (33.3)
Do you speak with a veterinary/livestock technician before using antibiotics in	
pigs? (*n* = 6)	
Yes	3 (50.0)
No	1 (16.7)
No response	2 (33.3)
Where do you normally get antibiotics?^a^ (*n* = 6)	
Agriculture Shop	3 (50.0)
Veterinary/livestock Technicians	2 (33.3)
Market	0 (0.0)
Pharmacy	0 (0.0)
No response	2 (33.3)
Have you ever stored antibiotics on your farm? (*n* = 6)	
Yes	2 (33.3)
No	2 (33.3)
No response	2 (33.3)
Do you record antibiotics used on your farm? (*n* = 6)	
Yes	0 (0.0)
No	5 (83.3)
No response	1 (16.7)
Do you wait for a few days after giving antibiotics before selling or slaughtering	
your pigs?	
Yes	2 (33.3)
No	2 (33.3)
No response	2 (33.3)

a *Multiple responses allowed*.

b *Penicillin G and streptomycin*.

c *Oxytetracycline*.

d *Sulphonamides +/– trimethoprim*.

For farmers whose pigs had been given antibiotics (*n* = 6), five used them to treat disease and one did not provide information on why antibiotics was used. None of the farmers reported a regular schedule of antibiotic administration to their pigs. One farmer reported that antibiotics had only been used once in pigs and four reported that antibiotics were used only when pigs were sick or in poor condition. The only reported route of administration of antibiotics was through injection (*n* = 5); no farmers reported that their pigs had been given antibiotics in feed or water. For those whose pigs were given antibiotics through injection, this was performed by the farmer themselves (*n* = 2) or by a government veterinary technician or extension worker (*n* = 3). Of the two farmers who injected their pigs themselves, one reported following label instructions during administration of the antibiotic. This farmer also reported preferring self-administration to sick pigs due to delays in veterinary technician arrival and the need to pay for private technicians. Another farmer procured antibiotics from an agriculture shop for the veterinary technician to perform the injection.

The commonly used antibiotics reported by farmers were oxytetracycline (*n* = 4) and sulphonamides (*n* = 1). The antibiotics were sourced from agriculture shops or veterinary technicians, and only those who sourced antibiotics from an agriculture shop had a history of storing antibiotics on their farms. No farmers kept written records of antibiotic use, and only two farmers reported waiting at least a few days after giving antibiotics before selling or slaughtering pigs.

## Discussion

This is the first study describing the knowledge of smallholder pig farmers in Timor-Leste on antibiotics and antibiotic resistance; and the antibiotic use practices in their pigs. It found that there was poor knowledge on antibiotics and antibiotic resistance, and that antibiotic use was very low among pigs belonging to smallholder pig farmers.

### Poor Knowledge on Antibiotics and Antibiotic Resistance

There was poor knowledge of antibiotics among farmers as most farmers said they did not know what antibiotics were and only one participant could correctly explain how an antibiotic worked. In addition, many farmers (23.0%) were unable to confirm if their pigs had received antibiotics because of their inability to distinguish antibiotics from other medicines or vaccines administered by injection. Furthermore, knowledge of antibiotic resistance was extremely poor as only one participant stated that they had heard of antibiotic resistance and the participant was unable to explain the concept correctly. This was unsurprising because no awareness or education campaigns on antibiotic resistance targeting farmers had been conducted prior to this survey (25). The poor knowledge of antibiotics and antibiotic resistance among smallholder pig farmers observed in this study was similar to Cambodia which is another LMIC in Southeast Asia (5, 37). A low level of knowledge was also reported in other LMICs, where only a minority of farmers raising livestock could explain antibiotics or antibiotic resistance accurately (28, 30, 38). Although sex, age, education level and location have been identified as factors influencing knowledge of antibiotic and antibiotic resistance (38–40), this study was unable to investigate these factors because of sample size and clustering at the suco level.

### Low Use of Antibiotics

The percentage of smallholder pig farmers who reported their pigs had been given antibiotics was very low at 3.6, or 6.1% if farmers whose sick pigs that received an injection from veterinary technicians were included. These estimates are much lower than in other LMICs in Southeast Asia where more than half of smallholder pig farmers reported that their pigs had received antibiotics (5, 41, 42). It would be interesting to compare antibiotic resistance levels in bacterial isolates from pigs in Timor-Leste to those from other LMICs (5, 43).

Possible reasons why very few farmers reported that their pigs had received antibiotics are the low input-output system of pig production in Timor-Leste (19) and the lack of access to veterinary services which was reported by the majority of farmers whose pigs have never received antibiotics. The lack of farmer access to veterinary services is consistent with other studies in Timor-Leste (11, 13, 19) and suggests that the provision of one veterinary technician per administrative post (25) is insufficient to cover all agricultural households in that area. However, there has been ongoing investment to improve farmer access to veterinary services over the last decade (25). It was interesting that Ritabou suco had the highest proportion of farmers whose pigs had been given antibiotics. This may be because of better antibiotic availability in an urban suco and its close proximity to Indonesia from where antibiotics are imported (25).

### Characterising Antibiotic Use Practices

Farmers in this study only reported sourcing antibiotics from veterinary technicians and agriculture shops which are stores where veterinary medicines can be obtained without a prescription. Studies in other LMICs also reported similar sources (30, 32), although additional sources such as human pharmacies and street vendors were mentioned (7, 29, 43). Most farmers whose pigs have received antibiotics sought advice from technicians prior to administration. Only one farmer reported not seeking advice. Studies in some LMICs show that most farmers sought advice from animal health professionals prior to administration (30, 42), while others show that farmers commonly use antibiotics without seeking prior advice from an animal health professional (5, 43) thereby possibly increasing the risk of antibiotic resistance due to misuse (44).

This study suggests that the use of antibiotics for disease prevention and growth promotion in pigs is uncommon among smallholder pig farmers in Timor-Leste. Farmers whose pigs had received antibiotics did not report routine usage. Rather, antibiotic use was only for treatment of sick pigs when the animals displayed clinical signs which were mainly respiratory and gastrointestinal. There was also no reported use of antibiotics via feed or water. Timorese farmers typically feed their pigs with a mix of household scraps, garden crops and/or rice bran cooked prior to giving to the pigs (19), which suggests that this response is likely genuine. The antibiotic products that were identified by farmers were also injectable antibiotics that are not indicated for growth promotion. Similarly, most smallholder pig farmers in Cambodia, Vietnam and Philippines reported using antibiotics only for treatment (5, 27, 32), but findings in other LMICs show that many farmers also use antibiotics for disease prevention or growth promotion (29, 39, 42).

The commonly used classes of antibiotics in pigs in this study (i.e., tetracyclines and sulphonamides) were also the most frequent antibiotic classes imported into Timor-Leste by the Ministry of Agriculture and Fisheries and agriculture shops (45). Tetracyclines are also the most common antibiotic class reported for use in food producing animals in Asia and the Oceania region (46). It is positive that none of the commonly used classes of antibiotics by smallholder pig farmers in Timor-Leste are classified as critically important antimicrobials (CIA) (47), in contrast to studies in other Southeast Asian LMICs that show common use of CIAs among pig farmers (5, 27, 42).

There is a public health risk of antibiotic residues in animal products when withdrawal periods are not followed (48). The findings from this study suggest that compliance with withdrawal periods is not routinely followed in Timor-Leste which is consistent with findings in other LMICs (5, 29, 41). Reasons for poor compliance to withdrawal periods in these studies include farmers selling sick animals early to avoid economic loss and the lack of regulation. In Timor-Leste, we suspect that the major reason for poor compliance to withdrawal periods is the lack of awareness because such a terminology does not exist in the vocabulary of Tetun. Future studies could investigate if farmers who are observing withdrawal periods are doing so because of residue risk or some other reason such as fear of zoonotic disease transmission from sick animals.

### Farmer and Farm Characteristics

Timorese pig farmers included in this study were similar to those included in other recent reports in some respects. Most participants in this study did not list pig rearing as a main income source. This is consistent with other studies which show that pigs are often a source of additional income and for cultural purposes (12, 13, 19). Farmer age and gender distribution was similar to two recent surveys of Timorese pig farmers (14, 19), except for more female farmers reported by Noronha (14).

However, some differences were apparent in pig management strategies. While this study found that very few farmers had free-roaming pigs, previous studies had found that allowing pigs to free-roam either all the time or during the day was very common (13, 14, 19). This change is likely due to high mortalities in free-roaming pigs coupled with increased awareness and efforts to house pigs to reduce the risk of ASF infection (49). Interestingly, Almeida et al. reported free-roaming management exclusively in rural sucos (19), but in this study some farmers in urban sucos indicated that their pigs were allowed to roam. The average number of pigs in this study was lower than reported elsewhere (11, 19). This is likely because of pig mortalities due to ASF since 2019 (22) which is consistent with reports by several farmers in this study who reported 100% mortality of their pigs.

### Strengths and Limitations

This study has several strengths. It addresses the lack of studies describing antibiotic use practices in LMICs (50) and can be used as baseline information to inform future interventions to combat antibiotic resistance. It will also support the implementation of the National Action Plan for Antimicrobial Resistance, specifically the objectives for improving awareness of antibiotic resistance and optimising the use of antibiotics (51). Non-response bias is limited as participation rate was very high. To reduce bias towards selecting farmers who had not been affected by ASF, this study included farmers who did not have pigs at the time of the survey but had raised them recently. Piloting of the questionnaire improved clarity and reduced potential for misclassification bias especially since it was designed in English but administered in Tetun. Finally, collection of relevant additional information on antibiotic use during the interview in free-text format allowed some understanding of the drivers of antibiotic use practices.

There were some limitations to the study. There may have been some bias in participant selection because the local guides may have introduced interviewers to farmers whom they were more familiar with. However, this was the most practical strategy given the lack of a sampling frame for households raising pigs at the suco level. Furthermore, such potential bias would be expected to result in an over-estimate of antibiotic use as such farmers would be expected to have better connexion to veterinary services. Separately, there may be a social desirability bias resulting in under-reporting of antibiotic use. However, this is expected to be limited considering the lack of knowledge about the negative impact of antibiotic resistance. Due to very low numbers of farmers reporting antibiotic use, only descriptive analysis was performed.

### Future Directions

The average herd size and intensity of pig production in Timor-Leste is expected to increase gradually because of ongoing agriculture developmental projects (52) and rising income levels which fuels demand for animal protein (15). This might increase antibiotic use for disease prevention and growth promotion among pig farmers to meet production goals, especially if there is no concurrent improvement to existing poor animal husbandry and farm biosecurity practices (19, 21). Furthermore, the lack of access to veterinary services for pig health issues may prompt farmers to seek their own interventions through self-administration of antibiotics, which increases the potential for misuse. This challenge has already been observed in other LMICs (29, 53).

Therefore, it is crucial to improve access to government veterinary services for farmers in Timor-Leste while disseminating knowledge on antibiotics and prudent use practices. This could be through farmer-targeted media campaigns and through technicians since they are a common source of knowledge and antibiotics for farmers in this study. Government-employed animal health professionals have also been identified as a trusted source of antibiotic use information for farmers in a study from Vietnam (54). The knowledge, attitudes, and practices of antibiotic use among technicians in Timor-Leste should be investigated since this is currently unknown and will help identify how technicians can be better equipped to promote prudent use practices among farmers (26, 55). Such a study can also identify any drivers of inappropriate usage among these animal health professionals that need to be circumvented, given drivers such as financial incentives have been reported by para-veterinarians in another Southeast Asian LMIC (56, 57).

## Conclusion

This study showed that the knowledge on antibiotics and antibiotic resistance is very poor among smallholder pig farmers in Timor-Leste. The low level of use of antibiotics in smallholder pig farming is reflective of the low input-output production system and limited farmer access to veterinary services. Among farmers whose pigs received antibiotics, it was positive that the purpose of use was exclusively for disease treatment and none of the antibiotics were CIAs. Farmer knowledge and practices on antibiotic use could be most effectively improved through knowledge dissemination via well-trained technicians since farmers identified them as a common source of antibiotic knowledge and supply.

## Data Availability Statement

The datasets generated in this study are available on request from the corresponding author.

## Ethics Statement

Ethics approval for this research was obtained from the University of Sydney Human Research Ethics Committee (Project number 2020/122). An information sheet was provided and verbally explained to all participants. Informed consent was obtained from participants through either a written signature or thumbprint. Participants were able to decline to participate or withdraw from the interview at any time.

## Author Contributions

ST, AP, J-AT, and JJ conceived and designed the study. AP and OM conducted the field work. ST, TB, AP, SD, AA, CS, and PS analysed the data. ST, TB, and SD prepared the draft manuscript. J-AT, JJ, AP, OM, AA, CS, and PS reviewed and edited the manuscript. All authors have read and approved the final manuscript.

## Funding

This study was supported by the Fleming Fund Country Grant for Timor-Leste (FF/17/233) and the Australian Centre for International Agricultural Research (ACIAR), through the small research activity Improved animal health surveillance in Timor-Leste (LS/2019/158). The Fleming Fund is a UK aid investment programme to tackle Antimicrobial Resistance in LMICs around the world and is managed by the UK Department of Health and Social Care.

## Conflict of Interest

TB is a working director of Epivet Pty. Ltd. The remaining authors declare that the research was conducted in the absence of any commercial or financial relationships that could be construed as a potential conflict of interest.

## Publisher's Note

All claims expressed in this article are solely those of the authors and do not necessarily represent those of their affiliated organizations, or those of the publisher, the editors and the reviewers. Any product that may be evaluated in this article, or claim that may be made by its manufacturer, is not guaranteed or endorsed by the publisher.
