# Supplementary material for: Knowledge and Practices on Antibiotic Use and Antibiotic Resistance Among Smallholder Pig Farmers in Timor-Leste
Source: Front Vet Sci. 2022 Jan 6;8:819643. doi: 10.3389/fvets.2021.819643 (PMC8770917; doi:10.3389/fvets.2021.819643)
Supplement: Supplementary file 1 [file Data_Sheet_1.PDF]

Knowledge and practice of antibiotic use and resistance

SMALLHOLDER PIG FARMER INTERVIEW

Section 1 – Demographics of participant and pig herd

Date of interview__________________ **Suco/Municipality** ______________________

Interviewer name ____

Respondent ID/Name____

1. Age____

2. □ Male □ Female

3. What is your education level? [Tick only one]

- □ Primary School
- □ Secondary School
- □ Post-secondary education
- □ No school
- □ Other (please describe) ____

4. How many pigs do you have at present? Write number of each pig type in the table

**Age**	**Total**
0-3 months	
4-12 months	
Sow	
Boar	
Castrated male	
**Total**	

5. Pig keeping methods [Tick only one]

- □ Free roaming all the time
- □ Tethered all of the time
- □ Housed all the time
- □ Housed with some free roaming or tethering
- □ Other ____

6. What is your main source of income? [Tick only one]

□ Pigs

□ Others: ____

Section 2 – Knowledge about antibiotics and antibiotic resistance

7. Do you know what antibiotic medicine for pigs are? [Tick only one]

□ Yes (If yes, where did you hear about them? (____)

□ No

If "No" - state that "Antibiotics is a medicine used to treat sick animals with bacteria infection" Then, proceed to question 11.

8. If yes, how do you think an antibiotic medicine works? [Tick all relevant]

Listen to answer and tick option/s described by farmer. Do not read list.

- □ Kill or inhibit virus
- □ Kills or inhibit bacteria [Correct answer]
- □ Kills or inhibit parasite
- □ Reduces pain (anti-inflammatory)
- □ Reduces fever (anti-pyretic)
- □ Other Please specify

____
____

If question 8 answer is wrong - state that "Antibiotics is a medicine used to treat sick animals with bacteria infection". Then, proceed to next question.

9. Have you heard of antibiotic resistance? [Tick only one]

- □ Yes (If yes, where did you hear about this ________________________________ ____)
- □ No

If "No" – skip to question 11.

10. If yes, what is the impact of antibiotic resistance? [Tick all relevant]
Listen to answer and tick option/s described by farmer. Do not read list.

- ◻ Antibiotic is less effective
- ◻ Antibiotic is more effective
- ◻ Other: Please specify ______________________________
- ◻ Don't Know

Section 3 – Practice of antibiotic use in pigs

It must be clearly established what is and is not an antibiotic before proceeding with this section.

11. Do you or anyone else give your pigs antibiotics? [Tick only one]

- ◻ **Yes**. If yes, how often? [Tick only one]
    - ◻ daily
    - ◻ weekly
    - ◻ monthly
    - ◻ every 3 months
    - ◻ once a year.
    - ◻ Other:______________________________
- ◻ **No**. If no, why. [Tick all relevant]
    - ◻ Expensive
    - ◻ Not available nearby
    - ◻ Not effective
    - ◻ Others: ______________________________
- ◻ **Don't know**

If answer to question 11 answer is No – ensure that understanding of antibiotic is accurate. If correct, then responses to question 12-23 is not required.

12. Why do you use antibiotics? [Tick all relevant]

- ◻ For treatment of diseases
- ◻ For prevention of diseases
- ◻ To promote growth
- ◻ Others: ______________________________

13. What signs in animals will prompt you to use antibiotic [Tick all relevant]

- ◻ Diarrhoea
- ◻ Fever
- ◻ Respiratory signs
- ◻ Skin infection
- ◻ Others: ____

14. Does your pig feed contain antibiotics? [Tick only one]

- ◻ Yes
- ◻ No

Comments:

15. Do you add antibiotics to water? [Tick only one]

- ◻ Yes
- ◻ No

Comments:

16. Have your pigs ever been injected with antibiotics? [Tick only one]

- ◻ Yes
- ◻ No

Comments:

17. Which antibiotics do you commonly use? [Tick all relevant]

- ◻ Penstrep
- ◻ Medoxy-LA
- ◻ Sulfra-strong
- ◻ Sulfabac
- ◻ Don't know
- ◻ others (especially if used in feed and water): ______________________________ ______________________________

18. Do you speak with a veterinary or livestock technician before using antibiotics in pigs? Either you or the technicians may be using it in your pigs [Tick only one]

- ◻ Yes (If yes, then why ______________________________ ______________________________)
- ◻ No (If no, then why ______________________________ ______________________________)

19. Do you follow the label instructions when using antibiotics? [Tick only one]

- ◻ Yes
- ◻ No (If no, ask for short explanation why ______________________________ ______________________________)
- ◻ Don't know (e.g. not the one that administers the antibiotic or can't remember)

Comments:

20. Where do you normally get antibiotics? [Tick all relevant]

- ◻ Agriculture shop
- ◻ Market
- ◻ Pharmacy
- ◻ Veterinary/livestock technicians
- ◻ Others: Please specify ______________________________

21. Have you ever stored antibiotics on your farm? [Tick only one]

- □ Yes (If yes, where?
  ____
  ____)
- □ No

Comments:

22. Do you record antibiotic used on your farm? [Tick only one]

- □ Yes. (If yes, how/where?
  ____
  ____)
- □ No

Comments:

23. Do you wait for a few days after giving antibiotics before slaughtering your pigs? [Tick only one]

- □ Yes. (If yes, why do you wait a few days?
  ____
  ____)
- □ No [“Sometimes” = No]

Comments:

